# Analysis of Microsatellite Variation in *Drosophila melanogaster* with Population-Scale Genome Sequencing

**DOI:** 10.1371/journal.pone.0033036

**Published:** 2012-03-12

**Authors:** John W. Fondon, Andy Martin, Stephen Richards, Richard A. Gibbs, David Mittelman

**Affiliations:** 1 Department of Biology, University of Texas at Arlington, Arlington, Texas, United States of America; 2 Virginia Bioinformatics Institute, Virginia Tech, Blacksburg, Virginia, United States of America; 3 Human Genome Sequencing Center, Baylor College of Medicine, Houston, Texas, United States of America; 4 Department of Biological Sciences, Virginia Tech, Blacksburg, Virginia, United States of America; 5 Department of Molecular and Human Genetics, Baylor College of Medicine, Houston, Texas, United States of America; Seoul National University College of Medicine, Republic of Korea

## Abstract

Genome sequencing technologies promise to revolutionize our understanding of genetics, evolution, and disease by making it feasible to survey a broad spectrum of sequence variation on a population scale. However, this potential can only be realized to the extent that methods for extracting and interpreting distinct forms of variation can be established. The error profiles and read length limitations of early versions of next-generation sequencing technologies rendered them ineffective for some sequence variant types, particularly microsatellites and other tandem repeats, and fostered the general misconception that such variants are inherently inaccessible to these platforms. At the same time, tandem repeats have emerged as important sources of functional variation. Tandem repeats are often located in and around genes, and frequent mutations in their lengths exert quantitative effects on gene function and phenotype, rapidly degrading linkage disequilibrium between markers and traits. Sensitive identification of these variants in large-scale next-gen sequencing efforts will enable more comprehensive association studies capable of revealing previously invisible associations. We present a population-scale analysis of microsatellite repeats using whole-genome data from 158 inbred isolates from the Drosophila Genetics Reference Panel, a collection of over 200 extensively phenotypically characterized isolates from a single natural population, to uncover processes underlying repeat mutation and to enable associations with behavioral, morphological, and life-history traits. Analysis of repeat variation from next-generation sequence data will also enhance studies of genome stability and neurodegenerative diseases.

## Introduction

Advances in genome technology are accelerating our understanding of the genetic basis for common traits and diseases. Large-scale efforts such as the HapMap Project have produced an initial catalog of genetic variants, primarily single nucleotide polymorphisms (SNPs), that has facilitated association studies with phenotypes [1]. The advent of accurate and cost-effective next-generation sequencing methods has now enabled the production of even more detailed maps of genetic variation. The 1000 Genomes Project and the Cancer Genome Atlas Project, for example, promise to illuminate genetic population structure and the genetic contribution to trait and disease phenotypes [2], [3]. However, an issue of missing heritability has been identified in many association studies, even for strongly heritable traits such as height [4]. The paucity of identified genetic determinants in genome-wide association studies may be partially explained by their reliance on high-frequency SNPs. At least in part due to technical limitations, the potential contributions of other forms of variation remains less fully explored [5].

Although much-used in the heyday of genetic linkage studies, polymorphic short tandem repeats, or microsatellites, were largely rendered obsolete as genetic markers by advent of genotyping microarrays, and are not broadly employed in GWAS [6]. However, tandem repeats continue to be broadly utilized as markers for genome instability and prognostic indicators for some forms of cancer [7], [8]. The roles of tandem repeats as causative agents of disease has been defined for a wide range of neurological and morphological disorders [9], [10], [11]. Furthermore, coding microsatellites are enriched prevalent in transcription factors and other regulatory proteins, where changes in repeat length exert incremental impacts on gene function [12], [13], [14]. Variations in the lengths of noncoding repeats in the promoters of genes have been shown to quantitatively affect transcription and can facilitate transcriptional plasticity [15]. Emerging evidence implicates coding and noncoding microsatellites as important sources of common genetic variation in morphological and behavioral traits in numerous species, including bacteria, yeast, flies, mice, dogs, and humans [16].

Despite the functional importance and unparalleled phylogenetic signal provided by tandem repeat variation, technical challenges have prevented its inclusion in the recent spate of “comprehensive” genomic variation analyses [5], [17]. Genotyping microsatellite repeats using next-generation sequencing is challenging for several reasons. At minimum, an individual read must span the entire repeat plus some flanking non-repetitive sequence for reliable local alignment and allele length determination. Furthermore, since repeats are abundant in most genomes, substantial additional unique sequence must be present in either the same read, or more commonly within its paired-end mate, to correctly map a repeat-containing read to the reference genome. The error spectra of some next-gen platforms further complicate the reliable ascertainment of repeat allele lengths. These issues extend beyond the well-known problems with mononucleotide repeats for the Roche 454 platform, affecting essentially all repeat types and platforms to some extent [18]. However, the advent of paired-end sequencing and increasingly longer read lengths are enabling more sensitive and accurate detection of structural variants and other problematic sequence variations [18], [19], [20].

Here we introduce a method to accurately genotype microsatellite repeats from next-generation sequencing data, and present a population-scale analysis of microsatellite repeats using assemblies of whole-genome Illumina data from 158 inbred isolates from the Drosophila Genetics Reference Panel [21]. These lines are a subset of nearly 200 extensively phenotypically characterized isolates from a single natural population from the Raleigh, North Carolina, USA area. First, we find that nearly a third of the 390,873 examined microsatellites are variable within this population, and confirmed a sample of these by Sanger sequencing. Next, we find that these polymorphic repeats generally conform to accepted models for repeat evolution in that repeat variation is predominantly in the form of insertions or deletions of whole repeat units, and polymorphism is correlated with repeat length and purity (i.e. fewer interruptions in the repeat sequence). These data help illuminate the processes underlying repeat mutation and will be instrumental in determining the contribution of repeats to quantitative variation in behavioral, morphological, and life-history traits.

## Results

### Length distribution of repeats in the *Drosophila melanogaster* reference sequence

Sequence read length determines the upper bound of repeat allele lengths that can be reliably determined by DNA sequencing. It is therefore useful to examine the distribution of repeat lengths in the finished *D. melanogaster* reference genome to estimate the proportion of microsatellite loci expected to be within reach of short read libraries. We identified all perfect and imperfect microsatellite repeats with a unit length of up to five nucleotides from build 5.13 of the *D. melanogaster* nuclear DNA reference sequence (see **Methods** section). About 12% of these microsatellites reside within or adjacent to larger repetitive elements, in heterochromatic regions, or in unscaffolded contigs to which reads cannot be uniquely mapped, and were excluded from further consideration (**Table 1**). Of a total of 390,873 microsatellite repeats satisfying minimum length and purity specifications, 92,047 (24%) were mononucleotides, 58,153 (15%) were dinucleotides, 95,234 (24%) were trinucleotides, 78,264 (20%) were tetranucleotides, and 67,175 (17%) were pentanucleotides. The median repeat length was 11 bases (range 7–651), and 90% of repeats were shorter than 23 nucleotides. Over 98% of microsatellites were accessible to the shortest reads employed in the DGRP sequencing libraries (45 bases), while only 165 repeats (0.04%) were beyond the reach of the longest reads (110 bases).

**Table 1:** Number of identified microsatellites and their association with repetitive elements by chromosome.

Chromosome	Total microsats	Number (%) in REs^a^
2L	103,467	6,444 ( 6)
2LHet	1,083	837 (77)
2R	92,291	7,306 ( 8)
2RHet	10,388	7,556 (73)
3L	114,997	7,719 ( 7)
3LHet	8,803	6,326 (72)
3R	127,212	4,328 ( 3)
3RHet	8,414	6,351 (75)
4	6,603	2,064 (31)
U	28,559	21,274 (74)
X	131,339	7,356 ( 6)
XHet	813	415 (51)
YHet	923	555 (60)

a Number (%) of microsatellites within 20 bases of a large repetitive element.

### Microsatellite genotype determination

The number and specific identities of deleted or inserted repeat units separating two different (or even identical) microsatellite alleles in a population is generally unknowable [22]. Genotyping tandem repeat variants in reference-mapped reads is therefore fundamentally distinct from calling SNPs or indels in non-repetitive sequence in that there is no sound basis for inferring homology between pairs of aligned repeat units. Therefore, microsatellite genotypes are scored in terms of allele length, or the number of sequenced bases within a read separating the non-repetitive flanking boundaries aligned to the reference, irrespective of intervening alignment gaps. Although separate reads of the same allelic variant might have been aligned with a gap/insertion at a different location within the repeat, the reads will all yield the same allele length call with this method. This approach effectively negates the well-known problem of large numbers of false positive SNP and indel calls resulting from inconsistent alignment of ambiguously positioned indels [18], [23], [24].

### Assessment of accuracy for genotype calls

We employed two metrics, completeness and internal concordance, to assess the comprehensiveness and accuracy of repeat genotype calls from whole-genome Drosophila data. The DGRP lines are each derived from single female founders of a natural fly population, and bred to near-isogeny by 20 generations of full-sibling matings. Therefore, although alleles may differ among lines, in the absence of mapping, alignment, or sequence errors, all reads from a single inbred line mapped to a specific microsatellite locus should possess the same repeat allele length. The assumption of homozygosity permits the use of internal concordance among the various reads within each inbred line to assess the relative accuracy of alternative approaches and tune heuristics:
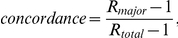
where R_major_ = the number of reads supporting the majority allele, and R_total_ = the total number of scorable reads at a repeat locus.

Regions of apparent residual heterozygosity were identified in individual lines on the basis of SNP genotypes, and were excluded from concordance assessments [21]. In conjunction with concordance, we employed another metric, completeness, or the proportion of repeats for which valid genotypes were obtained. The combination of these two metrics enables the evaluation of the relative accuracy and the comprehensiveness of various experimental approaches and heuristics.

To assign a genotype and assess concordance for a repeat, at least two scorable reads were required. A read was determined to be “scorable” on the basis of three criteria: First, the read must span the entire microsatellite and include flanking non-repetitive sequence on both ends. Second, a minimum number (initially, one) of consecutive flanking positions adjacent to the repeat must match the reference sequence. Finally, the read must have been uniquely mapped to the reference genome, with no alternative high-scoring hits to other regions of the genome.

### Most repeats can be genotyped using 75 base paired-end reads

The majority of the DGRP lines were sequenced using 45, 75, 95, 100, and/or 110 base reads to an average post-processed coverage of 21× [21]. The variety of read lengths employed presented a unique opportunity to investigate how read length impacts our ability to confidently assess repeat genotypes. We computed the concordance and completeness of microsatellite repeat genotypes as a function of the length of the repeat tract, as inferred from the allele length of the reference genome (referred to henceforth as reference length). The dataset included microsatellites for which at least 80% of bases in the repeat corresponded to perfect repetitions of the repeated unit (referred to henceforth as purity).

**Figure 1:**
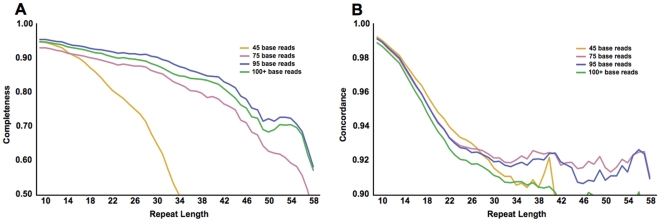
The completeness and internal concordance of microsatellite repeat genotypes in the Drosophila genome. The plotted values are the mean (A) completeness (fraction of repeats with at least two reads passing filtering criteria) and (B) concordance for the genomes in the DGRP panel, grouped by read length. Data have been smoothed for clarity (unweighted mean with window size ±2 bases). For this initial analysis, only a single matching base on each side of the repeat was required for a read to be scored.

For genomes sequenced using 45 base reads, about 50% of repeats with a reference length of 34 bases yielded genotypes and 3% of repeats with a reference length of 43 bases yielded genotypes (**Figure 1a**). In comparison, for genomes that were sequenced with 75+ base reads, an average 75% of repeats with a reference length of 43 bases yielded genotypes. In our dataset, 90% of the repeats had a reference length of 22 bases or less; and 45 base reads captured 79% of genotypes for repeats with a reference length of 22. Although 45 base reads yielded high-quality genotypes for most repeats in the Drosophila genome, the longest repeats tend to be the most variable, and so 45 base reads are unlikely to capture the majority of repeat variation in the DGRP lines.

Read length had only a modest impact on internal concordance. For read sizes of 45, 75, and 95 bases, the concordance of repeats at all reference lengths never fell below 90% (**Figure 1b**). The modest inverse correlation between read length and concordance observed for repeats shorter than ∼30 bases appears to result from the higher sequence error rates in later cycles of long read sequencing (data not shown).

**Figure 2:**
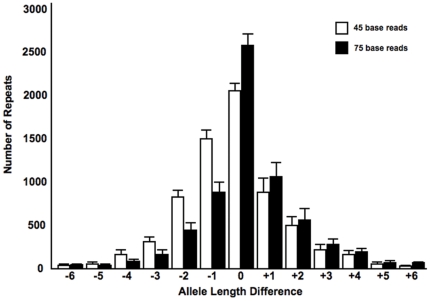
Analysis of discordant genotypes in genomes sequenced with two different read lengths reveals that short reads exhibit a bias towards shorter alleles. The difference between the inferred genotypes and the corresponding reference repeat length was tallied for 6,908 (out of 390,873) repeats for which different genotypes were obtained in the same inbred line from 45 base (open bars) versus 75 base (solid bars) reads. Permutation testing (1000 trials) indicates that the bias toward shorter alleles evident in the 45 base libraries is significant (for clarity, only the upper half of 95% confidence intervals are shown).

Some of the DGRP genomes were assembled from multiple libraries with different read lengths. In particular, there were seven genome assemblies possessing similar proportions of 45 and 75 base reads. These seven hybrid assemblies allow for direct comparisons of genotypes of the same individual derived from two read sizes. In these lines, an average of 263,994 (68%) repeats per line could be assigned genotypes using reads of both sizes. Of these, an average of 987 (0.4%) repeats per line yielded different genotypes between 45 and 75 base reads. These discordant loci exhibit a significant contraction bias in calls derived from 45 base reads, relative to the reference repeat length (**Figure 2**). The contraction bias in 45 base reads is most apparent for longer repeats (data not shown), consistent with bias in ascertainment of erroneously mapped (and gapped) reads. Since 45 base reads suffer from reduced completeness for longer repeats and significant contraction bias, they were excluded from subsequent optimization and benchmarking efforts.

### Genotype accuracy is affected by repeat length and type

Sequencing long microsatellite repeats is challenging and error-prone by any technology, including Sanger sequencing, with difficulties that extend beyond the known signal resolution limitations of the Roche 454 sequencing platform [18], [25]. Homopolymeric repeats are highly prone to *in vitro* slippage errors during polymerase-mediated replication, and are routinely masked for next-generation sequence analyses [26]. We therefore examined the contribution of repeat unit size to completeness and concordance, in order to determine unit size limitations for accurately measured changes in microsatellite repeats.

**Figure 3:**
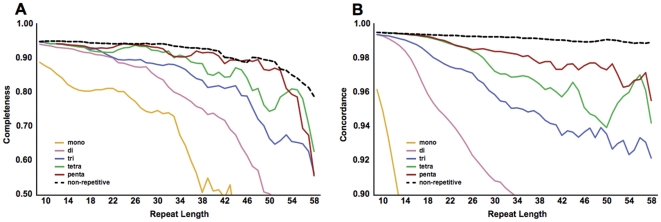
Repeats of shorter unit length are more difficult to sequence. The plotted values are the mean (A) completeness (fraction of repeats with at least two reads passing filtering criteria) and (B) concordance for the genomes in the DGRP panel. Data have been smoothed for clarity (unweighted mean with window size ±2 bases).

First, mononucleotide repeats were the least comprehensively genotyped repeat (**Figure 3A**). In contrast, pentanucleotide repeats were genotyped with the same level of completeness as matched non-repetitive regions. Second, internal concordance for mononucleotide repeats was considerably lower than for other repeats, falling below 0.9 for homopolymers longer than 13 bases (lengths based on the reference), and to 0.8 for repeats longer than 16 bases (**Figure 3B**). Completeness and concordance for longer repeat units were markedly better, with a mean concordance for dinucleotide repeats of at least 0.9 for repeats as long as 33 bases. The concordance for triplet repeats never fell below 0.92, and tetra- and pentanucleotide repeats never fell below 0.94.

We used Sanger sequencing to verify the lengths of 7 variable microsatellites, including GAGGG, ATACC, AC, A, T, and AAAT, in a total of 26 lines. These repeats were selected due to their association with startle response and starvation resistance in the DGRP lines [21]. Sanger sequencing confirmed the genotypes of all 26 genotypes derived from the Illumina data.

### Concordance is improved by filtering reads with flanking mismatches

Since regions of residual heterozygosity have been excluded, reads that span repeats in the inbred lines should all reflect the same repeat allele length. While recent de novo mutations cannot be excluded, discordant reads will predominantly be the result of errors in sequencing, mapping, or local alignment. Because microsatellites with similar or identical sequences occur at many locations in the genome, reads with repetitive sequences are more susceptible to misplacement with respect to a reference sequence. Manual inspection revealed improper mapping to be the predominant source of discordant reads. To reduce errors resulting from incorrectly mapped reads, we evaluated heuristics for selectively filtering reads exhibiting characteristics indicative of mapping error. First, we examined the relationship between concordance and mapping quality scores obtained from the mapping software. Like most short-read mapping programs, BWA assigns a Phred-like mapping quality score to each read (MapQ) based on match uniqueness, sequence identity, end-pairing, and inferred insert size, that is intended to indicate confidence of read placement accuracy [27], [28]. At shorter repeat lengths (10–24 bases) the mean MapQ value for reads mapped to a locus positively correlated with concordance (r^2^ = 0.99, p = 0.002). However, this correlation did not hold for repeat lengths greater than 24 bases (lengths 24–39, r^2^ = 0.65, p = 0.24; lengths 40–54, r^2^ = 0.57, p = 0.32). As a more sensitive test, we examined the MapQ of discordant singleton reads for loci with at least four reads supporting the majority allele (i.e. loci with allelic representation of *n*:1, with *n*≥4). Although the MapQ scores of discordant singletons were on average 10% below the mean of the majority reads at the same locus, the score distributions were not sufficiently distinct to support effective MapQ-based filtering. Similarly, although the distribution of base sequence quality scores declined more steeply toward the end of discordant singleton reads than majority reads, the overlap in distributions limits effective read filtering on the basis of sequence quality.

**Figure 4:**
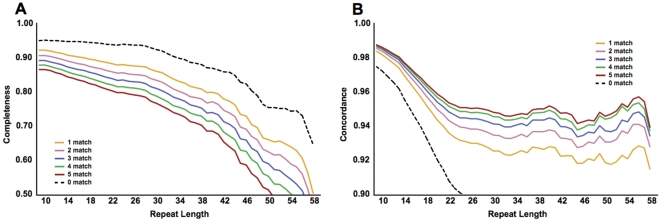
Increasing the requisite number of matching flank bases between a read and the reference improves genotyping accuracy at the expense of coverage. The minimum required number of matching flanking bases for a read to be scored was incremented from zero to five. Two or more scorable reads were required to determine a repeat genotype. The plotted values are the mean (A) completeness (fraction of repeats with at least two reads passing filtering criteria) and (B) concordance for the genomes in the DGRP panel. Data have been smoothed for clarity (unweighted mean with window size ±2 bases).

Manual inspection revealed that incorrectly mapped or aligned reads, and reads with poor sequence quality can often be identified by the presence of mismatches to the reference in the sequence immediately flanking the repeat. Increasing the minimum requisite number of consecutive perfectly matching flanking bases on both ends of the repeat resulted in modest drops in completeness (**Figure 4A**) but substantial improvements in concordance (**Figure 4B**). The improvement in concordance is exceeded by the loss in completeness when requiring more than three consecutive flanking matches.

### Properties of polymorphic microsatellite repeats

**Figure 5:**
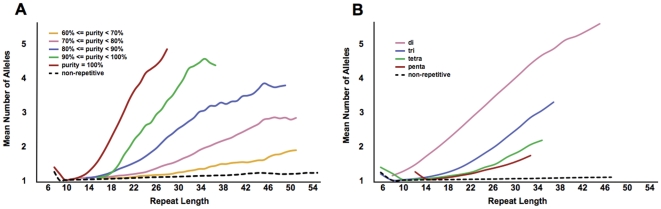
Variability is correlated with increasing repeat length, increasing purity, and decreasing unit size. Correlations were made using genotypes of repeats (2mers to 5mers) that were derived from genomes sequenced with a read length of at least 75 bases. Data points were plotted at each reference length bin interval that contained at least 25 repeats. The mean number of alleles (A) positively correlated with purity and (B) negatively correlated with unit size.

Almost any process that exposes single strands of DNA can lead to repeat length mutations, including replication, recombination, DNA damage repair, and other aspects of DNA metabolism [9], [29]. The susceptibility of a microsatellite to length mutations is largely a function of intrinsic properties of the repeat sequence, including the repeat unit length, the number of repeated units, and the purity of the repeat tract [30], [31]. In agreement with previous studies, we find that repeat tract length, purity, and unit size correlate with the average number of alleles for a repeat (**Figure 5**). The relationship between purity and length reveals that repeats possessing only one or two interruptions (**Figure 5A**, green line) evolve similarly to perfect repeats ∼6–8 nucleotides shorter (**Figure 5A**, red line), corresponding closely to the expected longest uninterrupted stretch of the imperfect repeats. However, a different dynamic emerges for more degenerate repeats, which exhibit step-wise decreases in slope with purity, yet all with similar intercepts. This pattern is not explained by uninterrupted segments of imperfect repeats, potentially suggestive of alternate mechanisms. In addition, dinucleotide repeats segregate from other repeats as the most variable (**Figure 5B**).

Since microsatellite length mutations almost always give rise to insertions or deletions of one or more whole repeat units, the minimum lengths at which short tandem repeats begin to exhibit this form of mutation can be determined by the emergence of excess unit-length variants over background mutation rates for nearby non-repetitive sequences. Makova and colleagues [32] recently used a related approach to delimit length thresholds for microsatellites within several regions Sanger sequenced in humans as part of the ENCODE project. In that study, the authors determined that human mononucleotide and dinucleotide repeats mutate above background slippage rates when the repeat tract is at least 10 bases [32].

**Figure 6:**
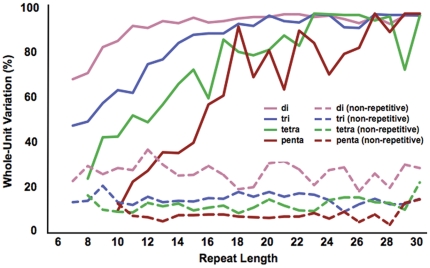
The tendency for differences in repeat length to occur in the form of insertions and deletions of whole repeated units increases with repeat tract length. The percent in-phase values of uninterrupted 2mer, 3mer, 4mer, and 5mer repeats approached a plateau at repeat lengths of 13, 20, 23, and 27 bases respectively, where length-changes are close to 100% in-phase. Genotypes for pure repeats were determined in all the DGRP lines if there were at least two scorable reads and a read was scored if it spanned the repeat region with 3 or more matching flank bases on either side of the repeat.

**Figure 7:**
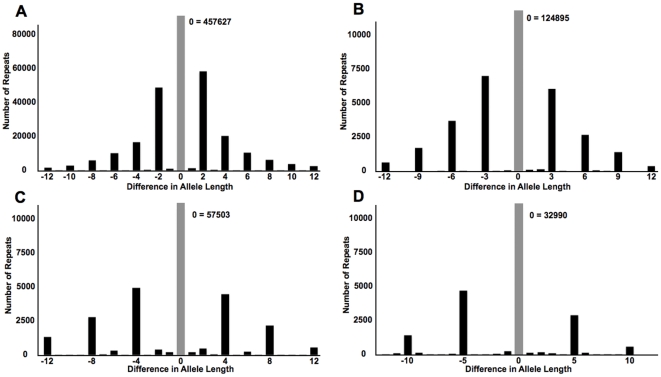
Changes in repeat length typically occur in the form of insertions and deletions of whole repeated units. The plotted dataset consisted of repeats that were at least 90% pure, with a minimum reference repeat length of 13, 20, 23, and 27 bases for (A) 2mers, (B) 3mers, (C) 4mers, and (D) 5mers, respectively. Genotypes were determined if there were at least two scorable reads and a read was scored if it spanned the repeat region with 3 or more matching flank bases on either side of the repeat.

We determined the lengths at which various repetitive sequences begin mutating as microsatellites by examining how the proportion of whole-unit variation to non-whole-unit variation changes as a function repeat length. We classified repeat variation from the DGRP lines in the form of the proportion of alleles that differ in whole-unit lengths from the most common allele relative to fractional unit length differences. As shown in **Figure 6**, we find that in *D. melanogaster*, the tendency for repetitive sequences to mutate in whole unit increments is clearly evident for even very short repeats. This tendency increases rapidly with tract length and eventually begins to plateau at approximately 13, 20, 23, and 27 bases for di-, tri-, tetra-, and pentanucleotide repeats, respectively. Most of the variation (97%, 96%, 82%, and 86%, for di-, tri-, tetra-, and pentanucleotides), in repeats at least as long as these plateau lengths, conforms to the classic step-wise model of microsatellite evolution (**Figure 7**). The majority of repeat lengths that were not whole-unit likely reflect errors in sequencing, mapping, or alignment. However, it is possible that some of this non-unit variation might also be indicative of other classes of indel mutations; or they might reflect instances of complex or imperfect repeats exhibiting mutational properties of multiple different units. Examples of the former possibility are most evident among tetranucleotide repeats, for which a large proportion of non-whole-unit variation is in multiples of two bases (**Figure 7C**). This half-unit excess is predominantly produced by imperfect repeats, but is also apparent in many perfect tetranucleotide repeats, suggestive of an alternative mutational process. Finally, although strand-slippage is expected to induce whole-unit mutations in uninterrupted repeats, repeats are also prone to double-strand breaks and if these breaks are not repaired by recombination-mediated processes, non-whole-unit changes to repeats can result [33], [34].

## Discussion

Nucleotide repeats are ubiquitous and polymorphic across all species. An often-cited example of physiologically and evolutionarily important microsatellite variation in Drosophila is a polymorphic threonine-glycine dipeptide repeat within the *period* gene. Naturally occurring length variation of the *period* coding repeat gene produces altered temperature-dependent circadian rhythm behavior in related populations of flies [35]. Natural selection has been demonstrated to act upon this locally adaptive variation, and it has been proposed that variation in such rhythm behavior underlies sympatric speciation events [36]. Furthermore, microsatellite repeats likely underlie the evolution of quantitative traits in many other species including mammals [16].

**Figure 8:**
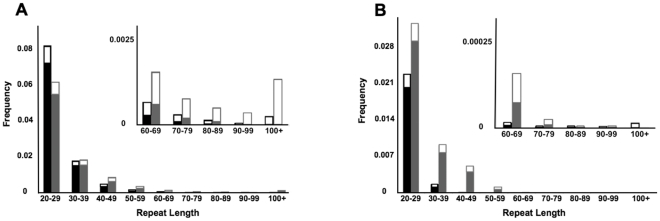
The distributions of repeat lengths in Drosophila and human genomes. The heights of the bars indicate the relative abundance of repeats at various lengths in Drosophila (black) and human (gray) genomes. The solid portions indicate the fraction of (A) all repeats 80% pure or greater, and (B) pure repeats that can be genotyped based on observed completeness using reads that are at least 100 bases.

We developed an approach to derive microsatellite repeat allele lengths from Illumina whole-genome data to gain insight into the mutational processes that modulate microsatellite variation and to enable the discovery of functional microsatellites. We find that that read sizes of at least 75 bases are sufficient to enable the accurate genotyping of most repeats in the *Drosophila melanogaster* genome and that mononucleotide repeats are the most challenging repeats to measure. Our proposed approach will gain even more utility for the ongoing data deluge as the read lengths for Illumina data now approach 150 bases. The approach can also be generalized to other genomes, including human genomes. Although the length distribution of microsatellites is longer in mammals than in Drosophila [37], repeats in normal human genomes, for example, almost never exceed 75 bases for pure repeats (**Figure 8A**) or even imperfect repeats (**Figure 8B**).

In the population of 158 inbred isolates from the Drosophila Genetics Reference Panel, we found that a third of the identified repeats vary in the population. Some of these repeats have already been related by GWAS to traits such as startle response and starvation resistance in the DGRP lines [21]. Undoubtedly, future studies with the DGRP population will reveal other associations between repeat length changes and trait variation. Next, we found that these polymorphic repeats follow accepted models for repeat instability—repeat mutation predominantly manifests itself in the form of insertions or deletions of whole repeat units and polymorphism correlates with increasing length and sequence purity. Finally, we used the DGRP dataset to ascertain the minimum lengths for a repetitive sequence to mutate as a microsatellite and find these values to be 13, 20, 23, and 27 bases for di-, tri-, tetra-, and penta-nucleotide repeats respectively.

While the above results establish a proof of principle that microsatellite repeats can be genotyped from short read next-generation sequencing data, the primary goal of this study is to catalog microsatellite variation in the DGRP lines to enable future studies of their contributions to variation in morphological, behavioral, and life-history traits. In the pursuit of this goal, all variants identified in this study are available online (http://genome.vbi.vt.edu/public/DGRP). A public web resource is also available to enable researchers to upload phenotypic data for association with microsatellite repeat variation, as well as other genetic polymorphisms (http://dgrp.gnets.ncsu.edu/). These resources will enable the scientific community to perform their own association studies and ultimately gauge the contribution of microsatellite repeat variation to quantitative traits in Drosophila.

## Methods

### Identifying microsatellite repeats from the Drosophila reference

Microsatellites were identified in the *Drosophila melanogaster* reference genome (release 5.13) using TRF v4.04 [38] using parameters “2 5 5 80 10 14 5,” and filtered to remove redundant hits. We excluded microsatellites within or adjacent to regions that preclude unique mapping, including larger repetitive elements and heterochromatin.

Transposons and other repetitive elements that confound short read mapping were identified using RepeatMasker (version 20071705; library release 20061006; -s setting). RepeatMasker results were filtered to remove all “Simple_repeat” and “Low_complexity” hits, and TRF-identified microsatellites occurring within 20 bases with of a RepeatMasker interval were removed. This reduced the microsatellite set from 634,892 regions to 556,361. A disproportionate number of the removed microsatellites were in heterochromatin and unscaffolded contigs (which are also mostly heterochromatin). We therefore chose to exclude the heterochromatic regions from analysis. The final set included 390,873 microsatellites.

### Mapping Illumina whole-genome from the DGRP lines

Methods for library preparation and sequencing are described elsewhere [21]. For the present study, we remapped all of the sequences for all 158 lines to the Dmel 5.13 reference genome using BWA (version 0.5.8c) with the “-n 5 –o 1 –e 3 –l 25” parameters [27].

### Microsatellite genotype inference

For each TRF-identified microsatellite, genotypes were scored by allele length, or the number of sequenced bases within a read separating the non-repetitive flanking boundaries aligned to the reference, irrespective of intervening alignment gaps. This approach ensures that insertions or deletions aligned to different portions of the repeat region in different reads are not scored as distinct alleles. Scripts and software used in the determination of repeat genotypes are available from the authors upon request.

### Correlations to length, unit size, and purity

To examine the relationships between unit size or purity and variability, genotypes of microsatellites of given unit sizes and purity values were analyzed to determine the number of unique alleles found within the DGRP dataset. The TRF-reported unit size and purity values were used to categorize the microsatellites by unit size or purity, while the most frequently observed allele length in the population was used for repeat length. Repeats were binned by length, and the mean number of distinct alleles for each bin was determined.

### Bootstrapping analysis

To evaluate the significance of allelic bias in 45 base versus 75 base libraries, 1,000 frequency distributions of allele length difference at discordant loci were created using microsatellites randomly sampled from the original set with replacement. For each allele length difference bin, the frequency values from each of these 1,000 randomized sets of repeats were sorted into increasing order and the 2.5^th^ and 97.5^th^ percentiles were plotted.

### Exclusion of residual heterozygosity

Regions of apparent heterozygosity within individual lines on the basis of heterozygous SNP genotypes were obtained from the DGRP project site [21]. Chromosomal arms were excluded from individual lines for concordance measurements if more than 5% of single nucleotide polymorphism sites were scored as heterozygous.
